# COVID-19 one year on: identification of at-risk groups for psychological trauma and poor health-protective behaviour using a telephone survey

**DOI:** 10.1186/s12888-022-03904-4

**Published:** 2022-04-09

**Authors:** Yuan Cao, Judy Yuen-man Siu, Daniel T. L. Shek, David H. K. Shum

**Affiliations:** 1 Department of Rehabilitation Sciences, The Hong Kong Polytechnic University, Hung Hom, Kowloon, Hong Kong SAR, China; 2 Mental Health Research Centre, The Hong Kong Polytechnic University, Hung Hom, Kowloon, Hong Kong SAR, China; 3 Department of Applied Social Sciences, The Hong Kong Polytechnic University, Hung Hom, Kowloon, Hong Kong SAR, China

**Keywords:** COVID-19, Coronavirus, Public health, Psychology, Trauma, Vaccine

## Abstract

**Background:**

This study examined the profiles and correlates of psychological trauma, compliance with preventative measures, vaccine acceptance and participation in voluntary testing during the novel coronavirus disease 2019 (COVID-19) pandemic among the adult population in Hong Kong (*n* = 3,011).

**Methods:**

Data were collected through a telephone survey between December 2020 and February 2021, using measures of psychological trauma, compliance with preventative measures, reading news reports on COVID-19, vaccine acceptance and willingness to participate in voluntary testing.

**Results:**

The prevalence of possible post-traumatic stress disorder was found to be 12.4%. Respondents were generally compliant with routine preventative measures, and approximately half had accepted vaccination and voluntary testing. Participants who had lower levels of education, were unemployed or had no income showed greater psychological trauma symptoms, whereas female, older and more educated participants showed greater compliance with preventative measures. Participants who spent more time watching news reports of COVID-19 had greater psychological trauma, but also greater compliance. Participants who were male, older, had lower education levels or were married showed greater acceptance of vaccination and participation in voluntary testing.

**Conclusions:**

Socio-demographic factors affected both psychological trauma and engagement in health-protective measures at one year after the onset of the pandemic. The theoretical and practical implications of these findings are discussed.

## Background

The unprecedented novel coronavirus disease 2019 (COVID-19) pandemic has created much psychological distress for people worldwide [1]. According to the World Health Organization (WHO), there have been more than 328 million confirmed cases of COVID-19, resulting in the deaths of more than five million people (as of 9^th^ January 2022; WHO live dashboard: https://covid19.who.int/). In many societies, people are required to follow official health guidelines (such as wearing masks and maintaining social distancing) to contain the spread of the virus. As the duration of the pandemic extends, there is a need to understand the mental health and health-protective behaviour of the general population sometime after the initial outbreak. In this study, we aimed to assess the psychological trauma and the effect of the pandemic on health-protective behaviour, including the practise of daily preventative measures, acceptance of vaccination and participation in voluntary testing after one year of the outbreak of the pandemic. Besides descriptive profiles, we also examined correlates of psychological trauma and health-protective behaviour during the COVID-19 pandemic in Hong Kong.

### Traumatic symptoms during the pandemic

Due to the forecasted long duration, constant news coverage and broad effects on all life domains, the COVID-19 pandemic has been more damaging than previous epidemics (e.g., severe acute respiratory syndrome [SARS]), and may be associated with more people showing symptoms of post-traumatic stress disorder (PTSD; [2]). The reported prevalence of PTSD related to the COVID-19 pandemic varies greatly in the international literature, from 8 to 50% [3], suggesting that the local context affects the psychological responses of citizens. In mainland China, Shek et al. [4] found that 10.4% of adolescents were suspected of having PTSD. In Hong Kong, Lau et al. [5] conducted a study (*n* = 761) between March and April 2020 using the Impact of Event Scale-Revised questionnaire [6], in which 28.6% of respondents were found to have probable PTSD. Other studies have shown that governments’ infection control policies have led to positive preventative behaviour outcomes at the cost of a negative impact on mental health [7, 8]. In Hong Kong, the societal and health contexts have changed in the past year as a result of the government’s implementation of COVID-19-related policies, including the implementation of two rounds of the Community Care Fund (first round, July–November 2020; second round, January–May 2021) for low-income persons or families and the introduction of vaccines. Therefore, there is a need to conduct updated research on the current prevalence rate of traumatic symptoms in Hong Kong to inform the government’s infection control policies for the further enhancement of preventative behaviour whilst balancing the need for the maintenance of good mental health. Second, we need to know the socio-demographic factors associated with relatively higher trauma symptoms, around one year after the start of the pandemic, to help with identifying the at-risk groups that need more assistance at this time. It is noteworthy that most of the studies on COVID-19 has been concentrated in the first year of outbreak.

Socio-demographic correlates of the mental health consequences of the COVID-19 pandemic have been reported in the international literature. For example, younger people (i.e., age) and females (i.e., gender) were found to have higher PTSD scores in mainland China [9] and in other countries, such as Spain [10]. However, other studies have found no correlation between age, gender or education level and PTSD scores (e.g., [11]). Conflicting results have also been reported in meta-analyses. For example, while Wang, Kala and Jafar [12] showed that women and younger adults are more likely to experience anxiety and depression related to COVID-19, Cenat et al. [3] found no significant effect of gender on anxiety or depression due to the pandemic. These conflicting findings suggest that further studies on the possible effects of age and gender on mental health are needed, particularly in non-Western contexts.

Furthermore, previous studies have shown that people who are socially disadvantaged, such as those with lower incomes, are more vulnerable to the physical [13], psychological [13] and social [13–15] effects of infectious diseases in general. In particular, an inadequate disposable income places a person at five times the risk of experiencing low subjective well-being than someone with an adequate income [16]. Given the large income disparity in Hong Kong (Gini coefficient = 0.539 in 2016, according to the Hong Kong Census and Statistics Department), it is important to determine whether economic disadvantage is related to PTSD symptoms during the COVID-19 pandemic. Previously, Lau et al. [5] reported a negative relationship between income and PTSD scores in the early stages of the pandemic.

Studies have also suggested that family conditions are associated with mental health. During the pandemic, caregivers of children with cognitive disabilities have experienced more anxiety and depression, compared with caregivers of children with ‘normal’ cognitive development [17]. However, there appears to be no or very limited large-scale research on how general caregiver status (e.g., carers of elderly individuals or family members with physical disabilities) affects psychological health during the pandemic. The role of marital status in this context also remains unclear, although marriage has been identified as a protective factor in the broader mental health literature (e.g., [18]).

Finally, the results of a meta-analysis suggested that, overall, greater media exposure is positively associated with increased psychological distress during the pandemic [12]. However, given that data from Hong Kong were not included in this meta-analysis, it is unclear if this finding applies to the special administrative region. The psychological reaction of Hong Kong residents to news reports about COVID-19 may have changed across the four waves of the pandemic. During the early stages of the pandemic, prolonged exposure to the news is associated with additional stress [12].

### Health-protective behaviour during the COVID-19 pandemic

A review suggested that the global rate of compliance with recommended COVID-19 preventative measures is between 25.9% and 98.8% [19]. Some studies on behavioural compliance have been conducted in the Hong Kong context. Based on a telephone survey of 3,013 adults in Hong Kong between January and March 2020, Cowling et al. [20] reported that approximately half of the respondents were worried about becoming infected with SARS-CoV-2, even though less than 25% of respondents felt that they were susceptible to infection. Moreover, approximately 75–99% of the respondents reported wearing a mask when leaving home, while only 61–90% avoided crowded places [20]. However, the demographic correlates of health-protective behaviour were not examined in that study. In April 2020, Zhao et al. [21] conducted telephone and online surveys of 1,501 participants in Hong Kong, and approximately 74% reported avoiding unnecessary outings, while only 59% avoided social gatherings.

While these two studies have contributed to our understanding of the views and behaviour of Hong Kong residents during the initial outbreak, there is a need to examine these issues further, because few studies have examined the situation one year after the start of the pandemic. More importantly, it is crucial to conduct a detailed analysis of the health-protective behaviour of people with different socio-demographic attributes. Two questions regarding health-protective behaviour during the COVID-19 pandemic need to be addressed. First, we need to obtain a more comprehensive and updated understanding of the health-protective behaviour of Hong Kong residents during the COVID-19 pandemic. This includes compliance with preventative measures, acceptance of vaccination and willingness to participate in voluntary testing. Second, we know little about the socio-demographic correlates of health-protective behaviour approximately one year after the start of the pandemic.

Previously, it was reported that, an older age, female gender and a higher educational level were associated with greater compliance with social distancing practices [19, 21]. The authors suggested that women and people with higher socio-economic status are more health conscious. Studying the effect of socio-demographic factors can help to characterise people’s preventative behaviours, and in turn, inform the policies and services related to the pandemic [22]. For example, in addition to population-wide campaigns, targeted campaigns may also be developed, to address the concerns of specific groups with lower compliances with infection preventions [22].

Family conditions may also affect the practice of preventative measures. A review by Wake [19] based on two international studies suggested that married people practise COVID-19-preventative measures more than unmarried people. However, being a caregiver of children is associated with less compliance with preventative measures, such as social distancing and personal protection [22]. Clinically, it can be reasoned that family caregivers have increased pressure to practise infection control to protect themselves and the person in their care [23]. However, few studies have explored how the family situation affects the practise of COVID-19-preventative measures, especially among carers of family members other than children (i.e., carers of elderly family members and children).

Finally, there is limited research data on the effect of media exposure related to COVID-19 on the practise of preventative measures in the general population. A study of pharmacists in Jordan found that media exposure was associated with increased risk perception, which was hypothesised to be a contributor to preventative practices [24]. This has also been reported during previous pandemics, such as the Middle East respiratory syndrome coronavirus pandemic (or MERS; [25]). We are unaware of any previous study assessing the effect of news-watching on health-protective behaviour in the general population during the COVID-19 pandemic.

### The present study

In this study, we examined Hong Kong residents’ psychological trauma (PTSD symptoms) and health-protective behaviour regarding COVID-19 infection at one year after the onset of the pandemic, with reference to the following research questions.

- What is the prevalence of psychological trauma among adults in Hong Kong during the COVID-19 pandemic, based on a large random sample?
- What are the socio-demographic correlates of psychological trauma during the COVID-19 pandemic? With reference to the literature, it was hypothesised that:
  - Hypothesis 1a: psychological trauma is positively associated with economic disadvantage [5], and;
  - Hypothesis 1b: watching COVID-19-related news reports is positively correlated with psychological trauma [2].
  - As previous research findings regarding the effects of gender, age, marital status and caregiver role on psychological trauma are not conclusive, no specific hypothesis was proposed for these factors.
- What is the level of engagement in health-protective behaviour amongst residents of Hong Kong?
- What are the socio-demographic correlates of health-protective behaviour during the COVID-19 pandemic? With reference to the literature, it was hypothesised that:
  - Hypothesis 2a: women display a higher level of health-protective behaviour [19];
  - Hypothesis 2b: age is positively associated with health-protective behaviour [21];
  - Hypothesis 2c: economic disadvantage is negatively associated with health-protective behaviour [21] and
  - Hypothesis 2d: married people are more compliant than unmarried people (e.g., [22]).
  - As previous research findings regarding the effects of caregiving and media exposure on health-protective behaviour are not conclusive, no specific hypothesis was proposed for these factors.

## Methods

### Participants

We aimed to recruit 3,000 participants which could give us representative responses based on the general adult population of Hong Kong (margin of error = 2%, population size of 6,413,800, 95% confidence level; [26, 27]).

Data collection took place between 18^th^ December 2020 and 2^nd^ February 2021. The target population was Cantonese-speaking Hong Kong residents aged 18 years and older. Inclusion criteria were therefore: a) respondents aged 18 years or above, b) Hong Kong residents, and c) people who spoke Cantonese. Data collection was performed by a local survey agency, using a computer-assisted telephone interview (CATI) system to call both landline and mobile phone numbers. Telephone numbers were randomly generated using known prefixes assigned to telecommunication services providers under the numbering plan provided by the Office of the Communications Authority (OFCA). For landline numbers, if there was more than one eligible respondent in the same household, the person whose birthday was the soonest or nearest to the date of the telephone survey was chosen as the participant for the survey. Calls took place between 6:30 p.m. and 10:00 p.m. on weekdays.

**Fig. 1:**
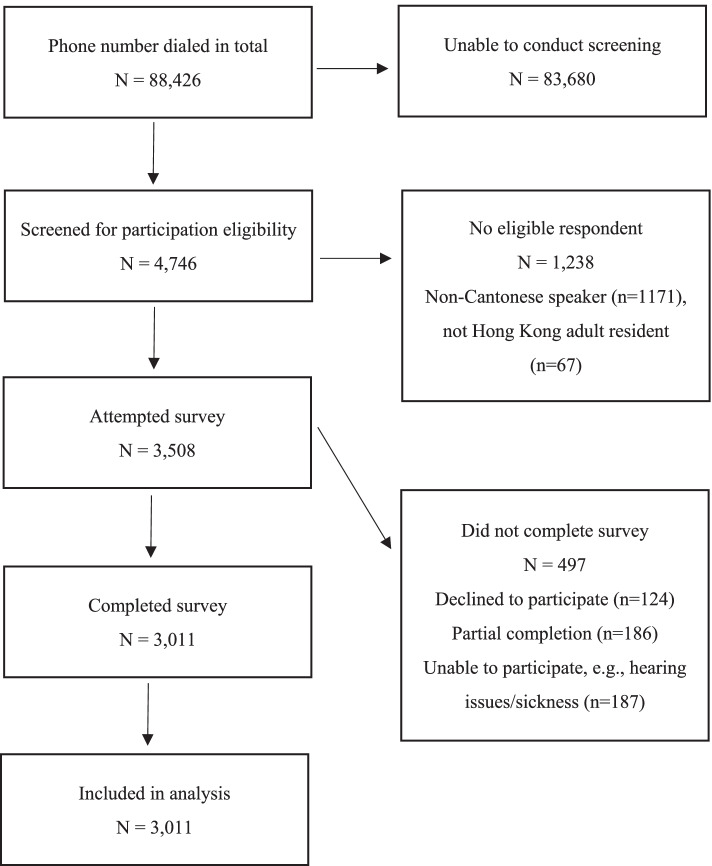
Flowchart showing numbers of individuals at each stage of study

Three thousand, five hundred and eight individuals agreed to participate in the survey after confirming their eligibility. Of these, 3,011 participants completed the full survey (85.8% response rate). The remaining 497 individuals either refused to take part or terminated the phone call prior to survey completion. The number of individuals involved at each stage is presented in Fig. 1.

The survey took an average of 18.2 min to complete (SD = 6 min). As the participants were allowed to decline to answer parts of the survey, there were some missing responses to the measures/demographic questions. The project received ethical approval from the Human Subjects Ethics Sub-Committee of the authors’ university.

### Measures

PTSD symptoms were measured using the *Revised Impact of Event Scale* (IES-R), a 22-item measure of the three main types of symptoms of PTSD: intrusion (8 items), avoidance (8 items) and hyper-arousal (6 items; [6]). This scale had previously been translated into Cantonese and psychometrically validated in Hong Kong (Cronbach’s alpha > 0.83; [28]). We sought permission from Dr. Wu for using the Cantonese translated version. It has been used in studies exploring the traumatic impact of COVID-19 locally [5, 11]. This scale asks about the presence and intensity of distress symptoms, in response to an event, which was defined to be the COVID-19 pandemic in the current project. An example item was “*I avoided letting myself get upset when I thought about COVID-19 or was reminded of it*”. The total score was used in this study, with higher scores reflecting more severe PTSD symptoms. Based on the results of previous studies, we used a cut-off score of 33 or above to indicate possible PTSD [5, 29, 30]. This scale also showed good reliability in the current study (Cronbach’s alpha = 0.860, 0.817 and 0.792 for the intrusion, avoidance and hyperarousal subscales, respectively).

We examined three types of health-protective behaviour in this study. The first behaviour was daily or routine practices, such as wearing face masks. For such daily health-protective behaviour, we used the *Questionnaire of Knowledge, Attitudes and Practice Towards COVID-19* (based on Zhong et al. [31]). We adapted this questionnaire for use in Hong Kong, with approval from the author Dr. Zhong. This questionnaire is a comprehensive measure that has previously been applied in the context of COVID-19 in Wuhan and internationally (e.g., in Malaysia and the U.S.). It contains questions examining the participant’s degree of compliance with the recommended prevention measures, such as regularly wearing face masks when outside the home, avoiding crowds, maintaining good hand hygiene, working from home and avoiding social gatherings. Table 1 lists all items included in the questionnaire. The questionnaire covers 14 preventative behavioural items scored using a 4-point Likert scale from 3 (always done this) to 0 (never done this). The average score, referred to as the Prevention Score in this paper, was used in the analysis. A higher score indicated greater compliance with the COVID-19 preventative health advice (Cronbach’s alpha = 0.762). In addition, we asked participants whether they had participated in voluntary testing and whether they were willing to receive the COVID-19 vaccine.

**Table 1:** Descriptive statistics on the Practice items of the *Questionnaire of Knowledge, Attitudes and Practice Towards COVID-19*

Behaviour^a^	Mean	S.D	Median	IQR	Mode
Wearing a face mask outside of home	2.965	0.201	3	3, 3	3
Reducing travelling	2.829	0.584	3	3, 3	3
Seeking medical advice if having signs of a respiratory infection	2.672	0.774	3	3, 3	3
Avoiding contacts with wild animals or their faeces	2.646	0.823	3	3, 3	3
Covering mouth with tissue when sneezing or coughing	2.623	0.726	3	2, 3	3
Putting the toilet lid down, before flushing	2.595	0.735	3	2, 3	3
Maintaining good environment hygiene by keeping good ventilation	2.535	0.65	3	3, 3	3
Reducing social gatherings	2.331	0.877	3	2, 3	3
Reducing going out or social outings	2.261	0.879	3	2, 3	3
Maintaining a social distance of 1-m from others as much as possible	2.208	0.885	2	2, 3	3
When washing hands, using water and soap, and rub hands for at least 20 s	2.206	0.863	2	2, 3	3
Cleaning hands using water and soap or hand-sanitizer before touching mouth, nose, or eyes	2.163	0.897	2	2, 3	3
Maintain drainage pipes properly and add water to the U-traps about once a week on average	2.023	1.037	2	1, 3	3
Work from home, or using flexible work hours	1.378	1.212	1	0, 3	0

^a^Ranked from highest to lowest compliance

Finally, we included questions to assess the participants’ level of secondary ‘exposure’ to the pandemic via watching COVID-19-related news reports (measured in minutes) and socio-demographic variables, including age, gender, education level, employment status, marital status, income level and family caregiver status (defined as providing long-term care for another member of the same household who was either elderly or had a disability). To be sensitive to the local culture, where directly asking age and income would be regarded as impolite, age and income were both collected as categorical variables. The item on the amount of time watching COVID-19 related news was “*In the last month, how long did you spend per day, on average, watching news relating to the COVID-19 pandemic?*”. This referred to watching news from any channel, including television or social media.

### Data analyses

Statistical analyses were performed using Statistical Package for Social Sciences (SPSS) version 26 (IBM, Armonk, NY, USA). Both descriptive and inferential statistical analyses were performed. More specifically, four logistic regression analyses were used to determine whether the demographic variables of interest were associated with PTSD (below/above the cut-off of 33), the Prevention Score (below/above median, representing daily health-protective behaviour), vaccine acceptance (yes/not yes, which included either unsure or no) and engagement in voluntary testing (yes/no). Logistic regression was used to analyse PTSD and Prevention Scores because these data were not normally distributed. More specifically, because the Prevention Score was strongly negatively skewed, we believed that the median is the most sensible cut-off point in the logistic regression analysis. The median point was 2.46, suggesting compliance with the preventative behaviours most of the time. We did not perform any imputations for missing values.

## Results

### Participant characteristics

The key characteristics of the 3,011 participants were: 53% female, 53.2% aged 30–59 years and 45.7% in full-time employment (Table 2).

**Table 2:** Participants’ demographic information

Demographic factor (total *n* = 3011)	n	% (of valid cases only)
Gender		
Male	1415	47.0
Female	1596	53.0
Age		
18–29 yo	474	16.0
30–59 (vs. 18–29 yo)	1579	53.2
60 or above (vs. 18–29 yo)	913	30.8
Missing	45	-
Education		
Primary school or below	564	18.9
Secondary school	1391	46.6
College or above	1028	34.5
Missing	28	-
Marital status		
Married	1785	60.4
Not married, i.e., Single/divorced/separated/widowed	1173	39.6
Missing	53	-
Work status		
Full-time	1370	45.7
Part-time	317	10.6
Unemployed (vs. full-time)	227	7.6
Retired (vs. full-time)	627	20.9
Student (vs. full-time)	136	4.5
Housemaker (vs. full-time)	320	10.7
Missing	14	-
Income		
No personal income	661	24.4
< HK$10,000	577	21.3
HK$10,000–19,999	599	22.1
HK$20,000–49,999	680	25.1
HK$50,000 or more	192	7.1
Missing	302	-
Carer status		
Is a carer	705	23.6
Not a carer	2281	76.4
Missing	25	-
Watch news		
Less than 15 min	589	21.5
15 – 29 min	384	14.0
30 – 59 min	745	27.2
1 h or more	1018	37.2
Missing	275	-

### Prevalence of psychological trauma

The results showed that 12.4% of respondents had a score ≥ 33 on the IES-R, suggesting possible diagnosis of PTSD (Table 3 and 4). Analyses of the average scores for the three subscales of the IES-R showed that 7.4% of participants had moderate levels of distress as indicated by the Avoidance and Hyperarousal subscales, and 8.1% showed distress as indicated by the Intrusion subscale (using a cut-off of 2 previously used in a Hong Kong study; [32]).

**Table 3:** Descriptive statistics for IES-R, Prevention Score and time spent on watching news

	Minimum	Maximum	Mean	S.D	Median	Mode
IES-R total score	0	88	16.05	13.94	12	4
Prevention Score	0.23	3	2.42	0.41	2.46	3
Time spent on watching pandemic news (min)	1	1440	58.09	96.233	30	30

**Table 4:** Distribution of participants on IES-R and Prevention Score using median split

	Lower than cut-off n (% of valid cases)	At or higher than cut-off n (% of valid cases)
IES-R total (lower or at/higher than clinical cut-off of 33)	2327 (87.6%)	330 (12.4%)

### Socio-demographic correlates of psychological trauma

Logistic regression analyses were performed to examine the possible effects of gender, age, educational attainment, work status, income, marital status, carer status and time spent watching news reports on the level of traumatic symptoms (*n* = 2,190). Multicollinearity was not a concern in this model (Gender, Tolerance = 0.933, VIF = 1.072; age, Tolerance = 0.603, VIF = 1.658; education attainment, Tolerance = 0.611, VIF = 1.636; marital status, Tolerance = 0.798, VIF = 1.253; work status, Tolerance = 0.514, VIF = 1.945; carer status, Tolerance = 0.982, VIF = 1.018; income, Tolerance = 0.486, VIF = 2.056; time spent watching news report, Tolerance = 0.962, VIF = 1.039). The logistic regression model was statistically significant, χ^2^(19) = 118.604, *p* < 0.0001, Nagelkerke *R*^2^ = 0.098 (Table 5). The Hosmer and Lemeshow test result was not significant, χ^2^(8) = 3.128, *p* > 0.05, suggesting a good model.

**Table 5:** Logistic regression on IES-R scores

Sociodemographic		Wald	df	p	OR	95% CI for OR
Gender	Male (vs. female)	2.070	1	0.150	0.814	0.614, 1.078
Age		6.584	2	0.037		
	30–59 (vs. 18–29 yo)	1.723	1	0.189	1.415	0.843, 2.376
	30–59 (vs. 60 or above)	5.201	1	0.023	1.686	1.076, 2.641
	60 or above (vs. 18–29 yo)	.267	1	0.605	0.839	0.432, 1.632
Education		6.403	2	0.041		
	Primary (vs. college or above)	4.698	1	0.030	1.696	1.052, 2.734
	Secondary (vs. college or above)	0.142	1	0.707	1.071	0.748, 1.535
	Secondary (vs. primary)	5.855	1	0.016	0.632	0.436, 0.916
Marital status	Married (vs. not married)	1.075	1	0.300	1.180	0.863, 1.613
Work status		12.321	5	0.031		
	Part-time (vs. full-time)	.376	1	0.540	0.856	0.521, 1.406
	Unemployed (vs. full-time)	4.525	1	0.033	1.838	1.049, 3.221
	Retired (vs. full-time)	0.080	1	0.778	1.087	0.610, 1.934
	Student (vs. full-time)	0.043	1	0.836	1.089	0.485, 2.447
	Housemaker (vs. full-time)	1.630	1	0.202	0.693	0.395, 1.216
Income		21.070	4	0.0003		
	No income (vs. HK$50,000 or more)	7.684	1	0.006	3.784	1.477, 9.697
	< HK$10,000 (vs. no income)	0.027	1	0.868	0.967	0.648, 1.442
	< HK$10,000 (vs. HK$50,000 or more)	7.548	1	0.006	3.658	1.450, 9.228
	HK$10,000–19,999 (vs. no income)	0.206	1	0.650	1.117	0.693, 1.798
	HK$10,000–19,999 (vs. HK$50,000 or more)	11.024	1	0.001	4.225	1.805, 9.892
	HK$20,000–49,999 (vs. no income)	6.336	1	0.012	0.482	0.273, 0.851
	HK$20,000–49,999 (vs. HK$50,000 or more)	1.957	1	0.162	1.824	0.786, 4.234
Carer status		1.947	1	0.163		
Watch news		35.509	3	< 0.0001		
	15 – 29 min (vs. less than 15 min)	0.023	1	0.880	0.959	0.559, 1.646
	15 – 29 min (vs. 1 h or more)	18.049	1	< 0.0001	0.376	0.239, 0.590
	30 – 59 min (vs. less than 15 min)	3.068	1	0.080	1.481	0.954, 2.299
	30—59 min (vs. 1 h or more)	10.997	1	0.001	0.581	0.421, 0.801
	1 h or more (vs. less than 15 min)	21.396	1	< 0.0001	2.551	1.716, 3.794

Middle-aged (i.e., 30–59 yo vs. 60 or above, odds ratio [OR] = 1.686, *p* = 0.023), lower educational attainment of primary school or below (vs. secondary school, OR = 0.632, *p* = 0.016; vs. college or above, OR = 1.696, *p* = 0.030), unemployment (vs. full-time employment, OR = 1.838, *p* = 0.033), lower personal income (no income vs. HK$20,000–49,999, OR = 0.482, *p* = 0.012; no income vs. HK$50,000 or above, OR = 3.784, *p* = 0.006; < HK$10,000 vs. HK$50,000 or more, OR = 3.658, *p* = 0.006; and HK$10,000–19,999 vs. HK$50,000 or more, OR = 4.225, *p* = 0.001) and spending more time (one hour or more) watching pandemic-related news reports (vs. less than 15 min, OR = 2.551, *p* < 0.0001; vs. 15–29 min, OR = 0.376, *p* < 0.0001; vs. 30–59 min, OR = 0.581, *p* = 0.001) were associated with possible PTSD.

Regarding the effect of income on IES-R score, post hoc comparisons (Table 6) also revealed that compared with the HK$20,000–49,999 group (*M* = 14.286, *SD* = 11.819), < HK$10,000 group (*M* = 16.622, *SD* = 14.163; *p* = 0.033) and HK$10,000–19,999 group (*M* = 17.119, *SD* = 14.713; *p* = 0.006) had a significantly higher IES-R score.

**Table 6:** Post hoc comparisons of the income groups on IES-R score

Group	*M(SD)*	Comparison group	Mean difference	*S.E*	*p*
No income	17.742 (15.617)				
		< HK$10,000	1.119	0.799	0.627
		HK$10,000–19,999	0.622	0.814	0.941
		HK$20,000–49,999	3.455*	0.784	0.0001
		HK$50,000 or more	6.164*	1.170	< 0.0001
< HK$10,000	16.622 (14.163)				
		No income	-1.119	0.799	0.627
		HK$10,000–19,999	-0.497	0.841	0.976
		HK$20,000–49,999	2.336*	0.812	0.033
		HK$50,000 or more	5.045*	1.189	0.0002
HK$10,000–19,999	17.119 (14.713)				
		No income	-0.622	0.814	0.941
		< HK$10,000	0.497	0.841	0.976
		HK$20,000–49,999	2.833*	0.826	0.006
		HK$50,000 or more	5.542*	1.199	< 0.0001
HK$20,000–49,999	14.286 (11.819)				
		No income	-3.455*	0.784	0.0001
		< HK$10,000	-2.336*	0.812	0.033
		HK$10,000–19,999	-2.833*	0.826	0.006
		HK$50,000 or more	2.708	1.179	0.146
HK$50,000 or more	11.578 (9.737)				
		No income	-6.164*	1.170	< 0.0001
		< HK$10,000	-5.045*	1.189	0.0002
		HK$10,000–19,999	-5.542*	1.199	< 0.0001
		HK$20,000–49,999	-2.708	1.179	0.146

Regarding the effect of time spend on watching pandemic-related news on IES-R score (Table 7), it was indicated that indicated that participants who spend 30–59 min watching pandemic-related news (*M* = 15.953, *SD* = 13.250) scored significantly higher in IES-R in comparison with those who watch 15 min or less (*M* = 13.674, *SD* = 11.849; *p* = 0.022).

**Table 7:** Post hoc comparisons of the time spend watching pandemic-related news groups on IES-R score

Group	*M(SD)*	Comparison group	Mean difference	*S.E*	*p*
Less than 15 min	13.674 (11.849)				
		15–29 min	-1.321	0.930	0.487
		30–59 min	-2.279*	0.794	0.022
		1 h or more	-4.987*	0.746	< 0.0001
15–29 min	14.995 (12.104)				
		Less than 15 min	1.321	0.930	0.487
		30–59 min	-0.958	0.886	0.701
		1 h or more	-3.666*	0.843	< 0.0001
30–59 min	15.953 (13.250)				
		Less than 15 min	2.279*	0.794	0.022
		15–29 min	0.958	0.886	0.701
		1 h or more	-2.708*	0.691	0.001
1 h or more	18.661 (15.848)				
		Less than 15 min	4.987*	0.746	< 0.0001
		15–29 min	3.666*	0.843	< 0.0001
		30–59 min	2.708*	0.691	0.001

### Engagement in health-protective behaviour

Participants had a mean Prevention Score of 2.42 (*SD* 0.41) and a mode of 3 (with 3 indicating ‘always done this’), suggesting that they often followed the health advice of the local government. Based on the means and medians of the 14 individual questionnaire items, there was relatively lower compliance with hand hygiene, working from home or using flexible work hours and pouring water into a U-trap approximately once a week (Table 1). The vaccine acceptance rate was 45.6% (Table 8), and more than half (56.4%) of the participants had participated in voluntary testing.

**Table 8:** Distribution of participants for the vaccine acceptance and participation in voluntary testing questions

	Yes n (% of valid cases)	No n (% of valid cases)
Vaccine acceptance	1359 (45.6%)	1618 (54.4%)
Voluntary testing	1696 (56.4%)	1313 (43.6%)

### Socio-demographic correlates of health-protective behaviour: daily practices

Logistic regression analyses were performed to examine the possible effects of gender, age, educational attainment, work status, income, marital status, carer status and time spent watching news reports on Prevention Scores/compliance with the recommended daily health-protective measures (*n* = 2,441). The model has an absence of multicollinearity (Gender, Tolerance = 0.925, VIF = 1.081; age, Tolerance = 0.604, VIF = 1.655; education attainment, Tolerance = 0.611, VIF = 1.638; marital status, Tolerance = 0.813, VIF = 1.231; work status, Tolerance = 0.512, VIF = 1.954; carer status, Tolerance = 0.983, VIF = 1.018; income, Tolerance = 0.491, VIF = 2.038; time spent watching news report, Tolerance = 0.960, VIF = 1.041). The logistic regression model was statistically significant, χ^2^(19) = 249.899, *p* < 0.0001, Nagelkerke *R*^2^ = 0.129; (Table 9). Hosmer and Lemeshow test result indicates a good fitting model, χ^2^(8) = 2.982, *p* > 0.05.

**Table 9:** Logistic regression on Prevention Score

Sociodemographic		Wald	df	p	OR	95% CI for OR
Gender	Female (vs. male)	49.102	1	< 0.0001	1.912	1.595, 2.292
Age		15.111	2	0.001		
	30–59 (vs. 18–29 yo)	9.229	1	0.002	1.616	1.186, 2.203
	30–59 (vs. 60 or above)	4.711	1	0.030	0.729	0.548, 0.970
	60 or above (vs. 18–29 yo)	14.934	1	0.0001	2.217	1.481, 3.321
Education		11.772	2	0.003		
	Secondary school (vs. primary)	10.973	1	0.001	1.565	1.201, 2.040
	Secondary school (vs. college or above)	0.181	1	0.670	0.953	0.765, 1.188
	College or above (vs. primary)	9.200	1	0.002	1.642	1.192, 2.262
Marital status	Married (vs. not married)	11.846	1	0.001	1.419	1.163, 1.732
Work status		10.498	5	0.062		
Income		4.472	4	0.346		
Carer status		0.309	1	0.578		
Watch news		28.749	3	< 0.0001		
	15 – 29 min (vs. less than 15 min)	16.597	1	< 0.0001	1.817	1.363, 2.423
	15 – 29 min (vs. 1 h or more)	0.002	1	0.967	0.994	0.767, 1.290
	30 – 59 min (vs. less than 15 min)	10.642	1	.001	1.505	1.177, 1.923
	30 – 59 min (vs. 1 h or more)	3.243	1	0.072	0.823	0.666, 1.017
	1 h or more (vs. less than 15 min)	25.811	1	< 0.0001	1.828	1.448, 2.306

Female gender (OR = 1.912, *p* < 0.0001), being a middle-aged (30–59 yo vs. 18–29-year-old adults, OR = 1.616, *p* = 0.002) or older adult (vs. 18–29-year-old adults, OR = 2.217, *p* = 0.0001; vs. 30–59-year-old adults, OR = 0.729, *p* = 0.030), an educational attainment of secondary school (vs. primary school, OR = 1.565, *p* = 0.001) and college or above (vs. primary school, OR = 1.642, *p* = 0.002), being married (vs. not married, OR = 1.419, *p* = 0.001) and spending more time watching news reports about the pandemic (vs. less than 15 min: 15–29 min, OR = 1.817, *p* < 0.0001; 30–59 min, OR = 1.505, *p* = 0.001; one hour or more, OR = 1.828, *p* < 0.0001) contributed to higher Prevention Scores.

Furthermore, post hoc comparison tests (Table 10) showed that compared with participants who watched less than 15 min (*M* = 2.297, *SD* = 0.412), those who watch 15–29 min (*M* = 2.434, *SD* = 0.367; *p* < 0.0001) and those who watch 30–59 min (*M* = 2.440, *SD* = 0.392; *p* < 0.0001) scored higher in Prevention Score.

**Table 10:** Post hoc comparisons of the time spend watching pandemic-related news groups on Prevention score

Group	*M(SD)*	Comparison group	Mean difference	*S.E*	*p*
Less than 15 min	2.297 (0.412)				
		15–29 min	-0.137*	0.026	< 0.0001
		30–59 min	-0.143*	0.021	< 0.0001
		1 h or more	-0.203*	0.020	< 0.0001
15–29 min	2.434 (0.367)				
		Less than 15 min	0.137*	0.026	< 0.0001
		30–59 min	-0.006	0.024	0.995
		1 h or more	-0.066*	0.023	0.023
30–59 min	2.440 (0.392)				
		Less than 15 min	0.143*	0.021	< 0.0001
		15–29 min	0.006	0.024	0.995
		1 h or more	-0.060*	0.018	0.006
1 h or more	2.500 (0.394)				
		Less than 15 min	0.203*	0.020	< 0.0001
		15–29 min	0.066*	0.023	0.023
		30–59 min	0.060*	0.018	0.006

### Socio-demographic correlates of health-protective behaviour: vaccine acceptance

Logistic regression analyses were performed to examine the possible effects of gender, age, educational attainment, work status, income, marital status, carer status and time spent watching pandemic-related news reports on the likelihood that participants would express willingness to receive the COVID-19 vaccine (*n* = 2,414). There was an absence of multicollinearity among the predictor variables (Gender, Tolerance = 0.927, VIF = 1.079; age, Tolerance = 0.605, VIF = 1.652; education attainment, Tolerance = 0.610, VIF = 1.640; marital status, Tolerance = 0.815, VIF = 1.227; work status, Tolerance = 0.513, VIF = 1.951; carer status, Tolerance = 0.984, VIF = 1.017; income, Tolerance = 0.490, VIF = 2.039; time spent watching news report, Tolerance = 0.960, VIF = 1.042). The logistic regression model was statistically significant, χ^2^(19) = 182.398, *p* < 0.0001, Nagelkerke *R*^2^ = 0.097 (Table 11). Hosmer and Lemeshow test result suggests a good model, χ^2^(8) = 10.117, *p* > 0.05.

**Table 11:** Logistic regression on vaccine acceptance

Sociodemographic		Wald	df	p	OR	95% CI for OR
Gender	Male (vs. female)	10.462	1	0.001	1.349	1.125, 1.616
Age		12.658	2	0.002		
	30–59 (vs. 18–29 yo)	5.926	1	0.015	1.496	1.082, 2.069
	30–59 (vs. 60 or above)	5.626	1	0.018	0.713	0.539, 0.943
	60 or above (vs. 18–29 yo)	12.599	1	0.0003	2.100	1.394, 3.162
Education		12.299	2	0.002		
	Primary (vs. college or above)	6.863	1	0.009	1.518	1.111, 2.075
	Secondary school (vs. primary)	0.080	1	0.777	0.964	0.745, 1.246
	Secondary school (vs. college or above)	11.691	1	0.001	1.463	1.176, 1.820
Marital status	Married (vs. not married)	23.223	1	< 0.0001	1.629	1.336, 1.987
Work status		4.976	5	0.419		
Income		2.539	4	0.638		
Carer status	Is a carer (vs. not)	4.975	1	0.026	1.252	1.028, 1.526
Watch news		7.683	3	0.053		

Male gender (vs. female gender, OR = 1.349, *p* = 0.001), being middle-aged (30–59 vs. 18–29, OR = 1.496, *p* = 0.015) or older (60 or above vs. 18–29 yo, OR = 2.100, *p* = 0.0003; vs. 30–59 yo, OR = 0.713, *p* = 0.018), a lower educational attainment (primary vs. college or above, OR = 1.518, *p* = 0.009; secondary vs. college or above, OR = 1.463, *p* = 0.001), being married (vs. not married, OR = 1.629, *p* < 0.0001) and being a family carer (OR = 1.252, *p* = 0.026) were associated with a greater willingness to receive the COVID-19 vaccine.

### Socio-demographic correlates of health-protective behaviour: voluntary testing

Logistic regression analyses were performed to examine the possible effects of gender, age, educational attainment, work status, income, marital status, carer status and time spent watching COVID-19-related news reports on participation in voluntary testing (*n* = 2,441). Multicollinearity was not a problem in this model (Gender, Tolerance = 0.925, VIF = 1.081; age, Tolerance = 0.604, VIF = 1.655; education attainment, Tolerance = 0.611, VIF = 1.638; marital status, Tolerance = 0.813, VIF = 1.231; work status, Tolerance = 0.512, VIF = 1.954; carer status, Tolerance = 0.983, VIF = 1.018; income, Tolerance = 0.491, VIF = 2.038; time spent watching news report, Tolerance = 0.960, VIF = 1.041). The logistic regression model was statistically significant, χ^2^(19) = 140.794, *p* < 0.0001, Nagelkerke *R*^2^ = 0.075; (Table 12). As revealed by Hosmer and Lemeshow test result, the model is good, χ^2^(8) = 5.787, *p* > 0.05.

**Table 12:** Logistic regression on participation in voluntary testing

Sociodemographic		Wald	df	p	OR	95% CI for OR
Gender	Male (vs. female)	1.637	1	0.201	0.889	0.743, 1.064
Age		8.779	2	0.012		
	30–59 (vs. 18–29 yo)	7.132	1	0.008	1.494	1.113, 2.007
	30–59 (vs. 60 or above)	1.108	1	0.293	0.857	0.644, 1.142
	60 or above (vs. 18–29 yo)	7.664	1	0.006	1.743	1.176, 2.583
Education		22.415	2	< 0.0001		
	Primary (vs. college or above)	6.881	1	0.009	1.517	1.111, 2.072
	Secondary school (vs. primary)	0.575	1	0.448	1.106	0.853, 1.435
	Secondary school (vs. college or above)	22.412	1	< 0.0001	1.678	1.354, 2.079
Marital status	Married (vs. not married)	17.371	1	< 0.0001	1.517	1.247, 1.845
Work status		12.688	5	0.026		
	Part-time (vs. full-time)	0.015	1	0.903	1.020	0.737, 1.413
	Unemployed (vs. full-time)	0.951	1	0.330	0.816	0.542, 1.228
	Retired (vs. full-time)	4.404	1	0.036	0.674	0.466, 0.974
	Student (vs. full-time)	3.996	1	0.046	0.592	0.354, 0.990
	Housemaker (vs. full-time)	8.144	1	0.004	0.584	0.404, 0.845
Income		1.248	4	0.870		
Carer status		0.281	1	0.596		
Watch news		0.536	3	0.911		

Being of middle-age (30–59 yo vs. 18–29 yo, OR = 1.494, *p* = 0.008) or older age (60 or above vs. 18–29, OR = 1.743, *p* = 0.006), having a lower educational attainment (primary vs. college or above, OR = 1.517, *p* = 0.009; secondary vs. college or above, OR = 1.678, *p* < 0.0001), full-time employment (vs. being retired, OR = 0.674, *p* = 0.036; a student, OR = 0.592, *p* = 0.046 or a housemaker, OR = 0.584, *p* = 0.004) and being married (vs. not married, OR = 1.517, *p* < 0.0001) were associated with greater participation in voluntary testing.

## Discussion

We performed a large-scale telephone survey to examine the level of traumatic response and compliance with preventative measures in the general population at one year after the beginning of the COVID-19 pandemic in Hong Kong. In addition, we focused on examining the socio-demographic correlates of PTSD symptoms, compliance with daily health-protective measures, willingness to receive the COVID-19 vaccine and participation in voluntary testing. As hypothesised, we found that socio-demographic factors, such as educational level, affected both psychological trauma and engagement in health-protective measures.

### Prevalence of PTSD

In terms of the prevalence of possible PTSD, 12.4% of participants received a score of ≥ 33 on the IES-R. Using the same scale and cut-off value, Lau et al. [5] found that 28.6% of adult respondents in Hong Kong between March and April 2020 showed possible PTSD. In a meta-analysis performed by Cenat et al. [3], the international prevalence of PTSD during the pandemic was found to range from 8 to 50%, with an average of 22% (based on studies published by May 2020). Therefore, the PTSD prevalence found in this study is within the range previously reported internationally. The lower PTSD prevalence in our study compared with the prevalence reported by Lau et al. [5] may be due to the timing of the study. Lau et al. [5] conducted their study soon after the community outbreaks occurred and as they started to worsen, whereas we collected our data during the ‘fourth wave’ of the pandemic. It is possible that people are becoming ‘numb’ in response to the uncertainty and uncontrollability associated with the continuous and repeated occurrence of local outbreaks [33]. Another possibility is that people have learned to cope better during the fourth wave, considering that it has been approximately one year since the initial outbreak. Most people are predicted to experience either no significant decline in their mental health or a recovery in their mental health with the progression of time after a traumatic event, and only a small percentage (approximately 5–10%) are predicted to show long-term distress [34]. The current rate of 12.4% is similar to this range, suggesting a possible similarity in recovery trajectory from the current pandemic to other traumatic events. However, considering the wide range of PTSD prevalence rates reported internationally, from 8 to 50% [3], and the rate of 28.6% reported in a previous local study [5], it is very likely that the mental health status is context-dependent to the disease and disease-management situation of the local city at the time of the survey, and so ongoing research is needed to explore the long-term impacts of the COVID-19 pandemic.

### Socio-demographic correlates of PTSD

This study also found that socio-demographic factors were related to the psychological response towards the pandemic. As hypothesised, people who were unemployed or had no personal income were found to have an increased likelihood of showing PTSD-like symptoms. This is consistent with existing research on the negative effects of low socioeconomic status on mental health [5, 35]. It has been suggested that the economic downturn due to the pandemic may have the greatest effect on those with a low income [5]. Therefore, assistance addressing the mental health issues of lower-income individuals is required, in addition to financial assistance.

No effect of gender on PTSD symptoms was observed in this study. This is consistent with the results of a recent meta-analysis by Cenat et al. [3]. Our results, therefore, suggest that other socio-demographic factors, such as employment and financial situation, are more important in determining an individual’s psychological experience of the pandemic. Similar to some previous studies [9, 10], there was a significant age effect. However, considering the inconsistency on such result in the existing literature [11], more research, especially meta-analysis, may be needed to better understand the effect of age on mental health during a pandemic. Unexpectedly, we also found no significant effect of family situation on PTSD scores. One possible reason is the definition of family carer used in our study. We identified people who were long-term carers of a family member (including children or elderly individuals), while Willner et al. [17] only included parents of children with cognitive impairments (who reported higher levels of anxiety and depression). Therefore, future research should explore whether different types of caregivers differ in their experience of the pandemic. This is warranted, as it has been reported that caregivers other than parents also experience increased pressure from the pandemic [23].

### Engagement in health-protective behaviour

There was an overall high level of compliance with health advice related to health-protective behaviour (Table 1). However, certain preventative measures were found to be more difficult to follow, including maintaining good hand-hygiene (washing hands for at least 20 s, and washing before touching the face) and environmental hygiene (pouring water into U-traps).

### Socio-demographic correlates of health-protective behaviour

Socio-demographic factors were also related to behavioural practices in relation to COVID-19 prevention. As hypothesised, middle-aged or older adults and people who were married were consistently more compliant with health advice related to preventative measures. This is consistent with the results of previous studies [21, 36]. In April 2020, it was found that older residents of Hong Kong self-reported greater compliance with social distancing measures during the pandemic [21]. In the broader health literature, being married or living with a partner is associated with healthier lifestyle choices, such as having health check-ups and avoiding alcohol and smoking [36]. One reason is that older and/or married people may be more health conscious [21]. Family carers were also found to be more willing to receive the COVID-19 vaccine, possibly due to a desire to prevent infection of the person in their care, considering that older adults are more prone to developing serious symptoms once infected [37].

Educational attainment was found to be positively correlated with daily health-protective behaviour (e.g., wearing a mask), but negatively correlated with vaccine acceptance and participation in voluntary testing. Higher educational attainment has generally been found to be a protective factor, associated with higher levels of health-protective behaviour during the COVID-19 pandemic in the international literature (see review by Wake [19]). In a systematic review based predominantly on U.S. studies, it was also concluded that higher educational attainment is associated with a greater acceptance of COVID-19 vaccines [38]. Our contradictory finding that people with a lower level of education tended to be more accepting of the COVID-19 vaccine replicates the findings of another study conducted in Hong Kong [39]. It is likely that, in Hong Kong, there are other reasons to explain this contradictory finding, such as the level of trust in health information [40]. More research is needed to understand why college graduates are less willing to receive the COVID-19 vaccine in Hong Kong. To overcome these issues, it has been suggested that clear health information should be disseminated via both formal and informal channels that are trusted locally, so that the message can be better received by the target audience [40]. Contrary to our expectations, we found no significant effect of income on daily health-protective behaviour, vaccine acceptance or participation in voluntary testing. This may be due to an issue with our sample, in that we had fewer participants with a higher income.

The effect of gender on behavioural practices also differed depending on the type of protective practice. For daily behaviour, such as wearing masks or hand hygiene, it was found that women reported better compliance with preventative measures, similar to previous studies. However, our results indicated that females were more likely to refuse the COVID-19 vaccine. This gender effect is also similar to results from other countries [41]. Vaccine acceptance has commonly been explained in the literature using the 3Cs model, where higher vaccine confidence and convenience and lower vaccine complacency have been found to contribute to increased vaccine acceptance (e.g., [42]). There is some evidence suggesting that women have less confidence in COVID-19 vaccines because they have more concerns about side effects [38]. The different attitudes towards vaccines versus the other prevention measures examined, such as hand-washing, could be due to the pharmaceutical vs non-pharmaceutical difference. A meta-analysis based on previous epidemics and pandemics showed that men have a higher willingness in adopting pharmaceutical health-protective behaviours compared to women [43]. However, as these are speculations only, more detailed research, such as in-depth qualitative studies, may provide us with further insight into the reason behind the gender difference, and the differing views on vaccine from other preventions. These results suggest that messages encouraging men to be more observant of daily health requirements and women to be more accepting of vaccines should be added to public campaigns about COVID-19 prevention.

### The effect of watching news reports on PTSD and health-protective behaviour

The amount of time spent watching pandemic-related news reports was found to be associated with more severe PTSD symptoms, but also with increased compliance with advice on daily preventative health measures. This is consistent with previous findings that over-exposure to pandemic-related news leads to psychological distress [12, 44]. To achieve a balance between disseminating important information and minimising distress to the audience, accurate and simple health messages need to be disseminated to the general public to increase people’s willingness to comply with preventative measures [9]. In addition, it may be helpful to suggest to the public that, to protect their mental health, they should avoid repetitively watching or listening to the same content [45]. Considering that there is a wide range of information sources from which one can obtain COVID-19-related news, the public should also be reminded to verify information before trusting it or sharing it with their own family and friends [39].

### Strengths and limitations

This study has several unique features. First, it was conducted one year after the initial COVID-19 outbreak. Hence, it provides an updated picture of the longer-term effects of the pandemic. Second, we evaluated both psychological trauma and health-protective behaviour, along with their socio-demographic correlates. In particular, we explored the relationships between COVID-19-related news consumption and psychological trauma and health-protective behaviour, which remain poorly understood internationally.

Results of this study should be interpreted in light of its limitations. This study aimed to determine ‘what’ is the level of PTSD and engagement in health-protective behaviour in the adult population. However, we have limited data to explain the reasons for the observed effects. Therefore, future research should continue to monitor the psychological state of the population and explore possible reasons underlying the observed changes. As this was a self-reported survey, we could not verify the accuracy of the answers related to the participants’ daily practices, or whether they had received the COVID-19 vaccine. Future studies should consider examining these questions in targeted groups, using collateral information from family members or health professionals. Related to this, we did not ask or check for the amount of time the participant spent on watching news in general, i.e., unrelating to the COVID-19 pandemic. This information would be useful for future studies to consider so that we can have better understanding of possible secondary exposure to the pandemic from watching relevant news coverage. The reported results represent the more recent situation in Hong Kong, approximately about one year after the start of the pandemic. However, a longitudinal study is needed to explore whether these patterns of behaviour will change with the future development or containment of the pandemic. Besides, due to the cross-sectional nature of this study, we must be cautious in making the claim that the level of PTSD is directly caused by the COVID-19 pandemic. Similar to previous discussions on this point, it is possible that the current results are reflective of a combination of the pandemic and the previous social unrest present in Hong Kong in 2019 [5]. We aimed to include a representative sample by performing random sampling when contacting potential participants. However, the sample was restricted to those who had a phone and were willing to participate, and the final sample included relatively few participants with a high income. Analyses were performed based on the responses given by the participants, with the possibility of a non-response (attrition) bias. Lastly, we are aware that the general population’s reactions or responses to the pandemic are dependent on the local context. Therefore, more studies in other countries are needed to better understand the correlates of the general population’s psychological state and behavioural compliance.

This study did not examine the possible underlying psychological mechanism, linking the pandemic with traumatic symptoms. However, this remains an important step for future studies. It is possible that people’s views and feelings relating to the future are disrupted due to the uncertain nature of both the COVID-19 disease itself and its possible impact on the world. Difficulties with accepting such uncertainty, or intolerance of uncertainty, could be a risk factor in increasing the chance of experiencing traumatic symptoms during the pandemic [46].

## Conclusions

This was a comprehensive, up-to-date study of the psychological trauma, compliance with preventative practices and related socio-demographic correlates in the general adult population of Hong Kong, approximately one year after the start of the pandemic. Using a telephone survey, we found that the prevalence of possible PTSD during the pandemic among the general population of Hong Kong was 12.4%. Respondents reported being compliant with daily preventative measures most of the time. The vaccine acceptance rate was 45.6%. Unemployment, a lack of income and lower educational attainment were associated with higher levels of psychological trauma. Female gender, older age, higher educational attainment and being married were associated with greater compliance with routine preventative measures. Male gender, older age, lower educational attainment and being married were associated with a greater willingness to receive the COVID-19 vaccine. The amount of time spent watching pandemic-related news reports was found to be associated with more severe PTSD symptoms and higher levels of compliance with daily health-protective measures. These results have implications for understanding the relationships between socio-demographic variables and health-related behaviour and mental health during a pandemic and for the design of governmental policies to help manage the effects of the pandemic and to prevent future outbreaks.

## Data Availability

The dataset generated and analyzed during the current study are not publicly available for participants’ confidentiality but are available from the corresponding author on reasonable request.
